# ERRATUM: Incidence of pressure ulcers in cardiopulmonary intensive care unit patients

**DOI:** 10.1590/1980-220X-REEUSP-2024-ER02en

**Published:** 2024-10-07

In the article “Incidence of pressure ulcers in cardiopulmonary intensive care unit patients”, with the DOI: https://doi.org/10.1590/S0080-623420150000700002, published by the journal: “Revista da Escola de Enfermagem da USP, volume 49 (spe) of 2015, p. 7–13, on page 13:

**Now read:**

## Financial support

This study was financed in part by the Coordenação de Aperfeiçoamento de Pessoal de Nível Superior – Brasil (CAPES) – Finance Code 001.

